# Influence of Potato Crisps Processing Parameters on Acrylamide Formation and Bioaccesibility

**DOI:** 10.3390/molecules24213827

**Published:** 2019-10-23

**Authors:** Emmanuel Martinez, Jose A. Rodriguez, Alicia C. Mondragon, Jose Manuel Lorenzo, Eva M. Santos

**Affiliations:** 1 Area Academica de Quimica, Universidad Autonoma del Estado de Hidalgo, Carr. Pachuca-Tulancingo Km. 4.5, Mineral de la Reforma, 42184 Hidalgo, Mexico; qaemmanuel@gmail.com (E.M.); josear@uaeh.edu.mx (J.A.R.); 2 Laboratorio de Higiene, Inspeccion y Control de Alimentos, Departamento de Quimica Analitica, Nutricion y Bromatologia, Facultad de Veterinaria, Universidad de Santiago de Compostela, 27002 Lugo, Spain; alicia.mondragon@deinal.es; 3 Meat Technology Centre of Galicia, Rúa Galicia Nº 4, Parque Tecnológico de Galicia, San Cibrao das Viñas, 32900 Ourense, Spain; jmlorenzo@ceteca.net

**Keywords:** acrylamide, crisps, temperature, frying time, blanching, thickness

## Abstract

A fractional factorial design was used to evaluate the effects of temperature, frying time, blanching treatment and the thickness of potato slices on acrylamide content in crisps. The design was used on freshly harvested and four-month stored potatoes. The critical factors found were temperature and frying time, and the interaction between blanching treatment and slice thickness. Once frying conditions were selected, an acrylamide content of 725 and 1030 mg kg^−1^ was found for non-stored and 4-month stored tubers, with adequate textural parameters in both cases. The difference in concentration is related to storage conditions, which must be controlled in order to control acrylamide levels. Bioaccesibility studies demonstrated that acrylamide concentration remained at 70%, and reductions took place mainly at the intestinal phase, as a result of reaction with nucleophilic compounds.

## 1. Introduction

Acrylamide, a thermal processing contaminant with a low molecular weight, which is soluble in water, is formed when carbohydrate-rich foods are subjected to temperatures above 120 °C in low-moisture conditions, such as frying, roasting or baking. Several studies consider Maillard reactions to be the main pathway for acrylamide, 5 hydroxymethylfurfural, methylglyoxal–lysine dimers, Nε–carboxymethyl–lysine and pyrraline formation in processed foods, particularly reactions between the carbonyl group of reducing sugars and amino acids [1,2]. Initial reports showed relatively high concentrations of acrylamide in high-carbohydrate foodstuffs, such as crispy bread, breakfast cereals, pastries, coffee, French fries and crisps. In general, potato products present higher acrylamide contents (250–4000 µg kg^−1^) compared to other food products, because of the higher concentration of asparagine in potato tubers [3,4]. 

Acrylamide has been classified by the Agency for Research on Cancer as “probably carcinogenic to humans” (Group 2A) and, in the latest report from the European Food Safety Authority Expert Panel on Contaminants in the Food Chain (CONTAM, 2015), the margins of exposure for acrylamide lead to concerns regarding their neoplastic effects, based on animal evidence [5]. Therefore, the European Commission recently established “indicative” levels for the presence of acrylamide in food, suggesting a limit of 750 µg kg^−1^ for crisps [6]. Previously, the European Union and FDA had encouraged food industries to reduce the presence of this contaminant.

In response to this, the food industry has been forced to apply different strategies to reduce acrylamide formation, applying processing modifications according to recommendations published by the European Food and Drink Federation in the document “Acrylamide Toolbox”, which includes the following variables: temperature and time during frying, blanching treatment, and thickness of potato slices [7]. However, the main challenge for the food industries is in producing potato chips with low acrylamide levels without affecting their sensory properties [8].

Although it is important to evaluate the effect of pre-treatments on the frying process on acrylamide mitigation, it is also necessary to determine their influence on its bioavailability. Potato chips are an important part of the snack food market in many countries, and few studies have been published about the bioavailability of acrylamide after crisps have been through the digestion process [8,9]. Acrylamide intake in humans occurs mainly via food ingestion, where the total amount of this compound does not necessarily reflect the available amount to the body. There are chemical changes in the food when it enters the digestive tract, because of pH variations, and the action of several enzymes in every stage of the digestion process [10,11,12]. In this sense, bioaccessibility is used to evaluate the amount of a chemical compound available for absorption after it is released from the food matrix into the gastrointestinal tract. Several methodologies can be used to assess the bioaccessibility of contaminants or nutrients: 1) in vitro, 2) ex vivo, 3) in situ and 4) in vivo models [13,14]. Among the techniques mentioned before, in vitro models are more commonly used than in vivo models, because of their simplicity, lower cost, lack of ethical issues and good reproducibility under controlled conditions.

The present work evaluated some acrylamide mitigation strategies, employing a fractional factorial design experiment, and their influence on textural properties and bioaccesibility, by an in vitro simulated digestion assay.

## 2. Results and Discussion

### 2.1. Acrylamide Content

Potatoes of the Atlantic variety were selected for crisp production, because of their low levels of glucose and fructose compared to other varieties, such as the Ranger Russet [15]. According to the storage period of potato tubers, two sampling points were considered: freshly harvested potatoes (summer of 2018) and four-month stored potatoes. The main variables described for acrylamide reduction in potato crisps are [7]: temperature control and time of frying process, blanching, and the thickness of slices. These variables were selected because they are easily applicable in the industry and do not represent extra costs in production. A fractional factorial design (2^4−1^) was proposed, to evaluate the influence of frying process parameters in the total amount of acrylamide formed and its bioaccessibility. The levels selected for the variables were: temperature (T) of 150 and 180 °C; frying time (t) of 5 and 10 min; blanching treatment (B)—no blanching treatment and 70 °C for 5 min—and thickness of slice (w), at 1.5 and 2.0 mm. In this sense, eight experiments (in duplicate, a total of 16 experiments) were performed. 

Table 1 shows the matrix used and the acrylamide content for the experimental design. According to the obtained results, the concentration of acrylamide was higher in stored potatoes. This can be explained by the difference between the content of reducing sugars between samples, since a concentration of 23 mg kg^−1^ of reducing sugars was found in freshly harvested potatoes, while, in samples stored for 4 months, a concentration of 34 mg kg^−1^ was found. During storage, a phenomenon called cold sweetening occurs, consisting of a degradation of the potato starch caused by the low temperatures [16]. Even if appropriate storage conditions of potato tubers are followed, a slight increase of reducing sugars is expected, and will affect the acrylamide concentration [17]. 

A comparison of each variance with respect to the variance of the residual allows the determination of the the experimental *F* value (*F_exp_*), which is then compared with the *F_critical_* value (5.32, *p* = 0.05, 1,8). The F-test indicated that, in both cases (non-stored and stored tubers), the critical factors for acrylamide formation were (*p* = 0.05): temperature (T) and frying time (t). The contribution of potato thickness (w) was also observed for stored samples (Table 2).

**Figure 1:**
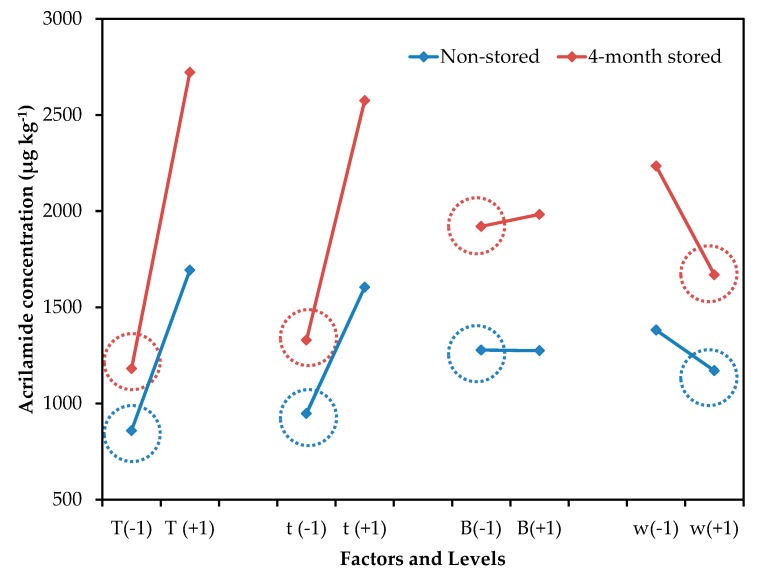
Mean effect graph vs. acrylamide concentration (µg kg^−1^) of the experimental design for non-stored and 4-month stored potato tubers storage.

Figure 1 shows the mean effect graph for the effect of evaluated control factors on acrylamide concentration. Independently of storage time, the mean effect profile is similar in both cases, demonstrating that the increase in acrylamide concentration is mainly a consequence of the increment of reducing sugars concentration, rather than the processing variables. Bertuzzi et al. also reported a strong influence of reducing sugar concentration on acrylamide formation in processed potato products [18]. A percentage of reducing sugars between 0.15%–0.20% has been suggested as an indicator of the suitability of potatoes for processing [19]. Despite the lower reducing sugar content found in potatoes in this study, acrylamide formation reached values over the 750 µg kg^−1^ limit established by the European Commission [6].

The results obtained are congruent with others previously described, in which an increment in acrylamide content in potatoes, in relation to temperature and frying time, was observed [18,20]. In general, the increase in acrylamide content follows a linear function over time, while the relationship with frying temperature is not linear, although temperatures over 175 °C significantly increased acrylamide levels [1,18,20,21,22]. Any temperature–time combination proposed to mitigate acrylamide content should not affect the overall quality of the fried product, especially its texture. 

**Figure 2:**
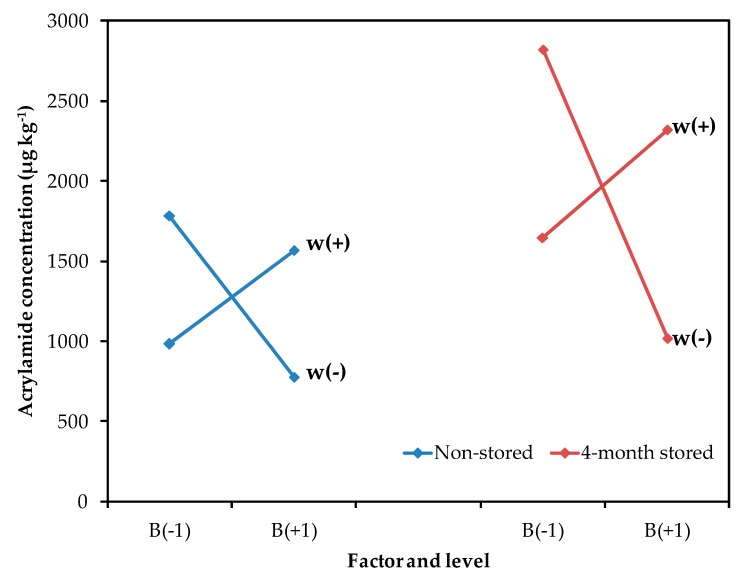
Mean effect graph vs. acrylamide concentration (µg kg^−1^) for the interaction between blanching (B) and thickness (w) in non-stored and 4-month stored tubers.

Although the other processing parameters (blanching and thickness) had a smaller contribution, their interaction was also significant (Figure 2). As acrylamide is formed on the potato surface, the size:volume ratio of the potato slice influences the acrylamide content. In general, thinner and smaller cut sizes result in increased acrylamide formation [22]. Also, the blanching of potato slides prior to the frying process has been recommended to reduce the acrylamide content, since this helps remove reducing sugars on the surface [8,23]. In this study, a blanching process was required for thinner potatoes, to decrease acrylamide formation. However, a lower concentration of acrylamide was observed in thicker potatoes without the blanching treatment. It seems that, in thicker slices, heat diffusion is slower when the slice has not been blanched and, consequently, a decrement of acrylamide content can be observed. So, the blanching treatment should not be suitable for potato slices with a thickness over 2.00 mm. This result is interesting for the industry because the application of blanching treatments at high temperatures and longer times represents an additional difficulty, since most industrial frying processes are continuous, and blanching could be unnecessary for certain types of crisps.

The combination of settings that generated the minimum acrylamide concentration was: temperature, 150 °C (−1); time, 5 min (−1); no blanching treatment (−1); and a slice thickness of 2.0 mm (+1). Acrylamide content of 725 and 1030 mg kg^−1^ was determined at these conditions for non-stored and 4-month stored tubers, respectively. 

### 2.2. Texture Analysis and Bioaccesibility 

**Figure 3:**
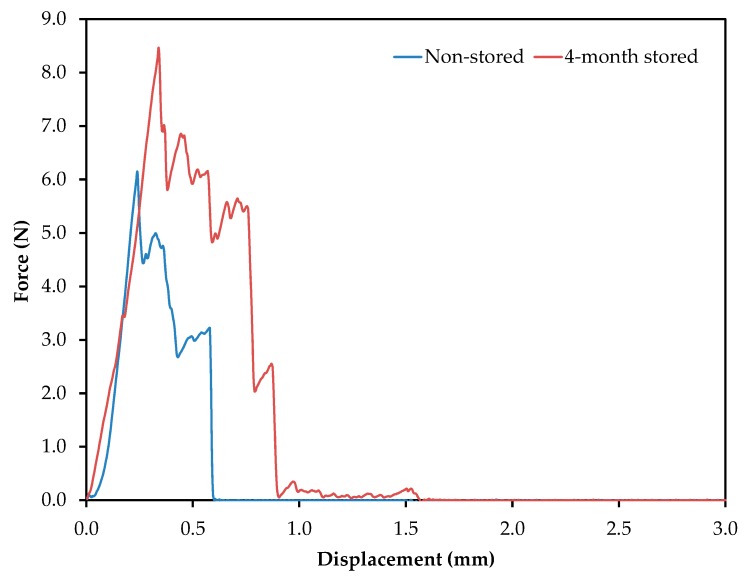
Force displacement curves generated from the analysis of potato crisps obtained from frying non-stored and 4-month stored tubers. Frying conditions: temperature, 150 °C; time, 5 min; no blanching treatment; and a slice thickness of 2.0 mm.

Potato chips are consumed as indulgent foods, with a characteristic flavor and crispiness, the latter evaluated through textural parameters. In order to evaluate it, crisps were prepared according to the processing parameters detailed above. Figure 3 shows a representative profile of the force (N) vs. probe displacement (mm). The force displacement curves have a jagged appearance, with several fracture events, which are typical of crispy food [24]. These graphics have two well-differentiated regions, the first one starting from the first contact between the potato crisp and the probe, until the major force is achieved (associated with major structural breakdown and hardness). The second region starts from the major structural breakdown and continues until the end, where smaller force events take place. The parameters evaluated were the maximum force applied (N), related to hardness or firmness, and the gradient (N s^−1^), which is related to stiffness. 

The maximum force applied was 6.1 and 8.5 N, for non-stored and 4-month stored samples, respectively. Genovese et al. have reported values below 6 N for firmness [25]. The storage of the tubers promoted an increase in firmness. On the other hand, samples obtained from frying the non-stored potato slices presented the highest gradient (8.9 N s^−1^) compared to 4-month stored samples (7.2 N s^−1^), making them, therefore, the stiffest. Additionally, the number of total force peaks in each case can be evaluated: seven for non-stored tubers and 13 for 4-month stored tubers. Taking into account that values higher than six are considered high-sensory crispiness, both samples can be accepted as adequate snacks [26].

Once the effect of the control factors on acrylamide formation had been evaluated, it was important to know their effect on bioaccesibility. Values of 79.1% ± 1.5% and 76.9% ± 1.5% of bioaccesibility were obtained for the snacks using potatoes that were freshly harvested and stored for 4 months before frying, respectively. The results obtained in this study are in agreement with the predicted behavior of potato [10] and bakery products [12]. The main effect was observed at the end of the intestinal phase of the digestion process, and could be explained by the formation of Michael adducts, in which the acrylamide would be involved.

During the gastric phase of the digestion process, pepsin hydrolyzes the proteins present in the potato, generating small peptide chains and some amino acid residues, such as cysteine and lysine, that have a nucleophilic character (-SH and -NH_2_). These peptides are able to interact with acrylamide and form adducts, which consequently causes an apparent reduction in acrylamide during the intestinal phase [12]. Taking into account the mentioned results, this can be proposed as an alternative method of reducing acrylamide concentration in the digestion process, through the formation of adducts with thiol compounds (i.e., glutathione) contained in other foods, such as spinach, avocados or asparagus [27,28]

## 3. Materials and Methods 

### 3.1. Reagents

Acrylamide, 2-naphthalenethiol, and the following enzymes: pepsin (≥250 U mg^−1^ solid), from porcine gastric mucosa, pancreatin (4 × USP), from porcine pancreas, α-amylase, from human saliva (500 U) and porcine bile salts, were purchased from Sigma–Aldrich (St. Louis, MO, USA). Potassium chloride, sodium bicarbonate and sodium chloride were purchased from MEYER (Mexico City, Mexico). Acetonitrile (HPLC grade) and acetic acid were obtained from J.T. Baker (Philisburg, NJ, USA).

### 3.2. Samples and Frying Conditions 

Potato (Atlantic variety) and vegetable oil (palm olein) were the raw materials used, provided by Fritos Totis (Tizayuca, Hidalgo, Mexico). Samples were collected in two periods, the first with potatos freshly harvested and the second after four months of storage, at 8 °C and 95% relative humidity. Slices (with a thickness of 1.5 and 2.0 mm, and a diameter of around 35 mm) were cut using a slicing machine. Slices were rinsed immediately after cutting for 1 min in deionized water (resistivity of 18.2 MΩ cm), to eliminate some starch material adhered onto the surface, before frying. Forty grams of slides were deep-fried in an electrical fryer Blazer FE-3 (Mexico city, Mexico) 1600 W, 127 V and equipped with a bowl with 3 L of capacity, a static basket and a regulating thermometer. 

The main variables evaluated for acrylamide reduction in potato crisps were [6]: control of temperature and time of frying process, blanching, and slice thickness. These strategies were selected because they are easily applicable to the industry and do not represent extra costs in production. A fractional factorial design (2^4−1^) was proposed, to evaluate the influence of frying process parameters in the total amount of acrylamide formed and its bioaccessibility. The factors selected were temperature (T), frying time (t), blanching treatment (B), and thickness of potato slices (w). Results obtained from the fractional factorial design were subjected to an analysis of variance study. The mean effect of each factor on acrylamide content and the variance was estimated through the Yates algorithm [29]. Experimental results were analyzed using MINITAB^®^ version 17 software (Minitab Inc., State College, PA, USA).

### 3.3. Acrylamide Determination

Acrylamide determination was carried out by chemical derivatization, using 2-naphthalenethiol as a derivatization reagent, followed by HPLC separation and quantification with fluorescence detection [30]. In brief, 3 g of potato crisps were homogenized with 30 mL of deionized water and then defatted with hexane. Then, the aqueous phase was centrifuged and filtered using a cellulose membrane (pore size, 0.45 µm). Afterwards, an aliquot of 7.5 mL was mixed with 1 mL of the solution 2-naphthalenethiol (62 mM), in a solution of sodium hydroxide (0.1 M). The mixture was heated at 90 °C for 45 min. The reaction was stopped with 1.5 mL of acetic acid (1%) and the solution was centrifuged at 700 g for 15 min. Acrylamide concentration of samples was calculated by standard additions, by spiking each sample with known concentrations of acrylamide. 

Chromatography separation was performed using an Agilent Technologies 1260 Infinity chromatography system (Agilent Technologies, Waldbronn, Germany). Samples were manually injected using a 20-µL loop, and a C-8 ZORBAX eclipse XDB column (5 µm; 150 × 4.6 mm internal diameter) from Agilent Technologies was used as the stationary phase. The mobile phase was acetic acid (1.0% *v*/*v*) and acetonitrile in a 50:50 ratio (*v*/*v*). The flow rate was 0.8 mL min^−1^. 

### 3.4. Texture Evaluation

A TA.XTPlus Texture Analyser (Stable Micro Systems Ltd., Surrey, UK), equipped with a 5 kg load cell, was used for force/displacement measurements, with a stainless spherical probe (P/0.25S) of a 0.25 inch diameter. The samples were placed on a crisp fracture rig (HDP/CFS). The test settings were: test speed of 1 mm s^−1^, trigger force of 0.406 N, travel distance of 3 mm. 

### 3.5. In Vitro Digestion 

Bioaccessibility was estimated using an in vitro digestion model (oral, gastric and intestinal stages) and the simulated fluids—salivary (SFS), gastric (SFG) and intestinal (SFI)—were prepared according to an internationally agreed protocol (Table 3) [31]. Samples of potato crisps (5 g) were transferred to a conical tube and mixed with 5 mL SFS (containing 75 U α-amylase mL^−1^) for 3 min. After that, 5 mL of a pepsin solution (12.5 mg mL^−1^ in 0.1 M HCl) and 10 mL of SFG were added. The mixture was adjusted to pH 2.0 and incubated in a shaking water bath at 37 °C for 2 h, with an agitation speed of 60 strokes per min. After incubation, the pH was adjusted to 7.5, and then bile salts (10 mg mL^−1^), dissolved in 20 mL of SFI together with a pancreatin solution (5 mL, 10 mg mL^−1^ in water), were added to the tube. The mixture was incubated at 37 °C for 2 h, with shaking at an agitation speed of 60 strokes per min, to simulate the duodenal phase. Digestion products were placed in a dialysis bag (6–8000 molecular weight cut off; Sigma Aldrich) and dialyzed in 250 mL of sodium bicarbonate solution (pH = 7.5) for 12 h. Dialysis aliquots were removed and acrylamide dialyzed was determined by the method described above. Bioaccesibility was reported as the percentage of acrylamide in the dialyzed sample compared to the value in the original sample.

## 4. Conclusions

The evaluation of potato crisps’ processing parameters was performed by a chemometric approach, through fractional factorial design. The factors with a higher contribution were temperature and frying time, and an increment on the acrylamide, with respect to the store time of raw material, was also observed. Crisps obtained in the most suitable conditions have adequate texture parameters, while bioaccesibility takes place mainly at the intestinal phase, through reaction with –NH_2_ and –SH containing compounds. In order to reduce acrylamide concentration in the digestion process, the consumption of foods which contain compounds with these functional groups is proposed.

## Figures and Tables

**Table 1:** Matrix of the experimental fractional factorial design and the average of obtained responses.

Exp	T (°C)	t (min)	B (70 °C, min)	w (mm)	Acrylamide Content (µg kg^−1^) ± sd	
					*Non-stored*	*4-month stored*
1	150(−1)	5(−1)	0(−1)	1.5(−1)	973 ± 71	1257 ± 171
2	150(−1)	10(+1)	0(−1)	2.0(+1)	752 ± 63	890 ± 52
3	150(−1)	10(+1)	5(+1)	1.5(−1)	825 ± 67	1483 ± 58
4	150(−1)	5(−1)	5(+1)	2.0(+1)	885 ± 62	1097 ± 205
5	180(+1)	10(+1)	0(−1)	1.5(−1)	2590 ± 243	4386 ± 270
6	180(+1)	5(−1)	0(−1)	2.0(+1)	795 ± 92	1149 ± 82
7	180(+1)	5(−1)	5(+1)	1.5(−1)	1138 ± 220	1812 ± 70
8	180(+1)	10(+1)	5(+1)	2.0(+1)	2250 ± 204	3540 ± 254

**Table 2:** Analysis of variance of the results obtained from the fractional factorial design, showing the factors significantly affecting acrylamide concentration.

Factor	Non-Stored			4-month Stored		
	Effect	Variance	F_exp_	Effect	Variance	F_exp_
T	834.5	2,785,561	**63.72**	1540	9,486,400	**216.99**
t	656.5	1,723,969	**39.43**	1246	6,210,064	**142.05**
B	-3	36	8.2 × 10^−4^	62.5	15,625	0.36
w	−211	178,084	4.07	−565.5	1,279,161	**29.26**
Residual		43,718			56,593.5	

**Table 3:** Composition of simulated salivary, gastric and intestinal electrolytes fluids.

Compound	Simulated Salivary Fluid (SSF) mmol L^−1^	Simulated Gastric Fluid (SGF) mmol L^−1^	Simulated Intestinal Fluid (SIF) mmol L^−1^
KCl	15.10	6.90	6.80
KH_2_PO_4_	3.70	0.90	0.80
NaHCO_3_	13.60	25.00	85.00
NaCl	-	47.20	38.40
MgCl_2_·6H_2_O	0.15	0.10	0.33
(NH_4_)_2_CO_3_	0.06	0.50	-
CaCl_2_·2H_2_O	1.50	0.15	0.60
